# Focusing on microglial mitochondria-lysosome crosstalk and neuroinflammation underlying depression: from molecular pathways to potential therapeutic interventions

**DOI:** 10.3389/fimmu.2026.1775841

**Published:** 2026-02-25

**Authors:** Xuelian Zou, Mingqin Shi, Xiangdian Xiao, Xiaoman Lv, Mengjia Yang, Miao Tian, Baiqing Xie, Lijuan Wang, Jing Wang, Dongdong Qin

**Affiliations:** 1 Key Laboratory of Traditional Chinese Medicine for Prevention and Treatment of Neuropsychiatric Diseases, Yunnan University of Chinese Medicine, Kunming, Yunnan, China; 2 First Clinical Medical College, Yunnan University of Chinese Medicine, Kunming, Yunnan, China; 3 School of Traditional Chinese Medicine, Qujing University of Medicine & Health Sciences, Qujing, Yunnan, China; 4 Department of Rheumatology and Immunology, Southern Central Hospital of Yunnan Province, Honghe, Yunnan, China; 5 Department of Rehabilitation, Kunming Children’s Hospital, Kunming Medical University, Kunming, Yunnan, China

**Keywords:** depression, lysosomes, mitochondria, mitochondria-lysosome contact sites, molecular pathways, neuroinflammation, therapeutic interventions

## Abstract

Depression is a prevalent emotional disorder that significantly impacts global health. Its etiology is multifactorial, and current therapeutic options have notable limitations, underscoring the need to identify novel molecular targets and therapeutic strategies. Neuroinflammation is a key pathophysiological feature of depression, with microglia serving as innate immune cells in the central nervous system (CNS), playing a crucial role in neuroinflammation sensing and amplification. Mitochondria and lysosomes, which are responsible for energy metabolism and waste degradation, respectively, forms non-fusogenic interactions at mitochondrial–lysosomal contact sites (MLCs) in microglia, promoting physical contact and signal transduction, thereby modulating microglial metabolic states and inflammatory phenotypes. Disruption of MLCs can lead to reactive oxygen species (ROS) accumulation, enhanced pro-inflammatory cytokine production, and amplification of neuroinflammatory cascades, thereby accelerating the neuroinflammation-driven pathogenesis of depression. In this review, we focus on how microglial MLCs drive neuroinflammation and contribute to the pathophysiology of depression. First, this review explores how peripheral immune dysregulation, oxidative stress, and impaired autophagy initiate and sustain neuroinflammatory responses that exacerbate depressive behaviors. Then, this review elucidates how mitochondrial dysfunction and lysosomal pathology amplify inflammatory signaling and promote the progression of depressive neurobiology. It highlights microglial MLCs abnormalities as a crucial mechanistic hub, detailing how disrupted Ca²^+^ crosstalk, impaired autophagic flux, and redox imbalance reinforce depression-related neuroinflammatory circuits. Finally, it summarizes emerging therapeutic strategies aimed at restoring microglial MLCs-regulated pathways and proposes future research directions to facilitate the development of neuroinflammation-targeted antidepressant therapies.

## 1 Introduction

Depression is a neuropsychiatric disorder mainly marked by long-lasting low mood, loss of interest or pleasure, and cognitive dysfunction (1). Depression is a major global public health problem. More than 400 million people are affected worldwide, with prevalence continuing to rise annually. By 2030, it is projected to be the leading mental health disorder, second only to cardiovascular diseases, in threatening human well-being (2–4). Current treatments include drugs, psychological therapies, and other physical treatments (5). However, even with many available antidepressants, about half of patients respond poorly, and nearly 30% show little or no improvement (6, 7). This underscores the urgent need for further research into the pathophysiology and therapeutic targets for depression. The limitations of current treatments, including delayed onset of action, side effects, and poor efficacy in treatment-resistant depression, highlight the need for novel molecular targets.

In recent years, researchers have increasingly focused on the pathogenic mechanisms of depression, particularly the critical role of neuroinflammation (8–10). Neuroinflammation is primarily mediated by glial cells, characterized by the release of pro-inflammatory cytokines. Glial cells form the brain’s immune system; when chronically activated or damaged, they overproduce pro-inflammatory mediators, exacerbating neuroinflammation (11, 12). Among these glial cells, microglia act as resident immune cells of the CNS, responsible for monitoring changes in the neural microenvironment and playing a crucial role in neuroimmune responses to injury or activation (13–15). In microglia, mitochondria and lysosomes coordinate energy metabolism and waste clearance (16, 17). Recent studies have demonstrated that these organelles interact via mitochondrial–lysosomal contact sites (MLCs), forming a critical signaling network that regulates cellular functions (18, 19). Beyond supporting energy production and macromolecular turnover, MLCs regulate autophagy, redox homeostasis, and apoptosis (20). At these contact sites, MLCs promote autophagic flux, modulate redox responses, and regulate ion exchange, thereby shaping microglial function (21, 22). The altered functions of MLCs may influence neuroinflammation’s onset and persistence, thereby contributing to depression.

This review synthesizes current evidence on neuroinflammatory mechanisms in depression, with an emphasis on microglial contributions. We further highlight microglial MLCs as a mechanistic hub linking organelle crosstalk to neuroinflammatory signaling and depression progression. This perspective may inform the identification of therapeutic targets aimed at modulating neuroinflammation and restoring microglial homeostasis.

## 2 The neuroinflammatory mechanisms of depression

A key feature of neuroinflammation related to depression is the persistence of pro-inflammatory signals and the failure of inflammation resolution (23, 24). Microglia, the resident innate immune cells of the CNS, are central to sensing, integrating, and amplifying inflammatory responses (25, 26). Peripheral immune dysregulation, oxidative stress, and autophagy-lysosomal dysfunction can promote the sustained pro-inflammatory phenotype of microglia and maintain the pro-inflammatory cascade (27–29). Collectively, these intertwined mechanisms contribute to the initiation and progression of depression.

### 2.1 Peripheral immune dysregulation and neuroinflammation

The immune and nervous systems exhibit bidirectional crosstalk via neural and humoral pathways (23, 30). Peripheral inflammatory signals can affect the CNS through the signal transduction or transport mechanisms of blood-brain barrier (BBB)-related structures (5, 31, 32). Many studies have shown that chronic immune-mediated inflammation plays an important role in the development and progression of depression (24, 33, 34). Proposed mechanisms include altered monoamine neurotransmission (35), changes in neurotrophic factor expression, impaired synaptic plasticity, and induction of neuroinflammation associated with depressive-like behaviors (36–38). Studies by Haapakoski et al. (39) and Su et al. (40) reported associations between depression and elevated inflammatory cytokines in cerebrospinal fluid. In the CNS, cytokines are mainly produced by microglia, astrocytes, neurons, and other immune cells (12, 41). Peripheral inflammatory mediators cross the BBB and influence the CNS, activating glial cells and triggering local cytokine release. This process, facilitated through MLCs, thereby forming a positive feedback loop and exacerbating neuroinflammation (12, 31, 42). Cytokines may further promote depressive symptoms by shunting tryptophan metabolism toward the kynurenine pathway and disrupting hypothalamic–pituitary–adrenal (HPA) axis feedback (5, 23, 43–45).

### 2.2 Oxidative stress triggers neuroinflammation

Beyond immune activation, oxidative stress is a key driver of neuroinflammation, a core pathological process implicated in depression (46, 47). Oxidative stress arises when ROS generation exceeds cellular antioxidant capacity, resulting in redox imbalance and downstream molecular injury (48). Many studies have shown that depression is linked to reduced antioxidant activity (49, 50). Oxidative stress damages lipids, proteins, and DNA and can amplify ROS production by activating NADPH oxidase 2 (NOX2) (51, 52). Increased ROS can elevate intracellular Ca²^+^ levels through pathways such as ROS-sensitive ion channels or abnormal calcium handling in organelles, thereby enhancing inflammatory signal transduction (53). Excess ROS may also compromise BBB integrity and facilitate peripheral-to-central immune signaling, thereby sustaining neuroinflammation and synaptic dysfunction (23, 54). In microglia, chronic oxidative stress can impair mitochondrial bioenergetics and disrupt endolysosomal and lysosomal degradation, amplifying a feed-forward ROS–inflammation loop (55, 56). This coupling favors the persistence of neuroinflammation. In chronic social defeat stress models, elevated microglial ROS is associated with neuroinflammatory activation and has been proven to be involved in driving depressive-like behavioral phenotypes (57, 58).

### 2.3 Dysfunction of autophagy triggers neuroinflammation

Autophagy is a physiological cellular stress response that removes damaged organelles and misfolded proteins via lysosomal pathways to keep cellular homeostasis (59–61). Clinical and experimental studies indicate that autophagy dysregulation contributes to depression pathogenesis, in part by shaping neuroinflammatory signaling (62, 63). Altered autophagy markers have been reported in patients with depression and in animal models, including within microglia (62, 64). Disruption of the autophagy–lysosome pathway raises the inflammatory set point. When autophagic flux is impaired, damaged mitochondria and pro-inflammatory substrates accumulate, increasing ROS and danger-associated signaling, and promoting the initiation and amplification of inflammatory cascades (65–67). Notably, autophagy plays a crucial role in suppressing NLRP3 inflammasome activation and limiting the release of IL-1 family cytokines (65, 68–70). Recent studies have shown that during depression, microglial autophagic flux is specifically regulated at MLCs, with key proteins like PINK1 and LC3 being persistently dysregulated in depression models (71–73). This dysregulation leads to defective mitophagy, which further exacerbates neuroinflammation. Accordingly, reduced autophagy can permit sustained activation of inflammatory pathways and accumulation of pro-inflammatory cytokines, thereby exacerbating neuroinflammation and promoting depression-relevant pathology (74). Autophagy also intersects with Ca²^+^ signaling. Impaired autophagy can perturb Ca²^+^ homeostasis, heighten microglial inflammatory reactivity, and ultimately affect emotional and cognitive functions (75).

## 3 The role of mitochondria and lysosomes in the neuroinflammation underlying depression

Disrupted energy metabolism and impaired cellular clearance are increasingly recognized as convergent pathophysiological features of depression (76–78). Mitochondria and lysosomes are important for cellular energy production and waste degradation (79, 80). Studies have shown that these organelles do not function in isolation. Instead, they communicate through MLCs, which coordinate mitochondrial dynamics and ion and metabolite handling to shape cellular metabolism (18, 81). Under the context of depression, dysfunction of these organelles, particularly microglial impairment, plays a critical role in mediating neuroinflammation and impacting neuronal viability (76–79).

### 3.1 Microglial mitochondrial dysfunction

Microglial mitochondrial dysfunction is widely recognized as a key molecular correlate of neuroinflammation in depression (82, 83). In depressed states, microglial mitochondria undergo alterations in number, morphology, and electron transport chain activity, accompanied by mutations and deletions in mitochondrial DNA (84). Collectively, these abnormalities reduce mitochondrial membrane potential and ATP output while promoting excessive ROS generation (85, 86). Excess mitochondrial ROS acts as a pro-inflammatory signal by engaging redox-sensitive pathways, thereby activating microglia and increasing pro-inflammatory cytokine release, which aggravates neuroinflammation (24, 76). Neuroinflammation can then impair synaptic transmission and further disrupt mitochondrial function, creating a feed-forward loop that amplifies oxidative stress and inflammatory signaling and ultimately worsens depressive phenotypes (24, 76). Furthermore, chronic stress can hinder the clearance of dysfunctional mitochondria by mitophagy by inhibiting the stability of PTEN-induced kinase 1 (PINK1) and its accumulation on damaged mitochondria, a process that leads to the continuous accumulation of damaged mitochondria, further amplifying oxidative stress and chronic neuroinflammation (87). These pathological disturbances (oxidative stress, neuroinflammation, and mitochondrial dysfunction) significantly impair neurotransmission (88), thereby exacerbating depression-related symptoms (89, 90).

### 3.2 Microglial lysosomal dysfunction

In addition to mitochondrial impairment, microglial lysosomal dysfunction is a critical contributor to neuroinflammation underlying depression (80). Lysosomes degrade intracellular macromolecules and serve as signaling hubs that couple nutrient sensing to mTORC1 activity. Through mTORC1-dependent control of TFEB nuclear localization, lysosomes also regulate lysosomal biogenesis and downstream homeostatic programs (91–93). Under depressive conditions, lysosomal activity is often markedly impaired. Studies report that key microglial lysosomal proteins—including lysosomal-associated membrane protein 1 (LAMP1), cathepsin D, and TFEB—are downregulated (94–96). These alterations can impair lysosomal acidification and hydrolase activity, thereby weakening the degradation of damaged cellular components (97–99). This, in turn, compromises autophagic flux. Specifically, defects in lysosome–autophagosome fusion can occur alongside reduced acidification and lysosomal protein expression, leading to the accumulation of dysfunctional organelles and proteins. This buildup impairs microglial clearance capacity (including phagocytic processing) and can promote neuroinflammatory signaling (100, 101). Additionally, lysosomal dysfunction prevents the efficient degradation of defective organelles and misfolded proteins. The resulting accumulation of danger-associated molecular patterns (DAMPs) can promote NLRP3 inflammasome assembly and activation, thereby sustaining IL-1 family cytokine signaling (102). Impaired autophagy and reduced lysosomal activity in microglia disrupt neuronal homeostasis and worsen neuroinflammation, exacerbating depressive symptoms.4 Microglial MLCs.

It has been clarified in the previous text that mitochondrial and lysosomal dysfunction in microglia is an important pathological feature in neuroinflammation-associated depression. The functional synergy and signal transmission between the two organelles depend on the specific MLCs formed between them. As a non-fusogenic inter-organelle communication hub, MLCs affect the maintenance of homeostasis and inflammatory responses of microglia. Their functional abnormalities can exacerbate neuroinflammation through multiple mechanisms and promote the occurrence of depression.

### 3.3 Structural characteristics and physiological functions of MLCs

MLCs in microglia are increasingly recognized as important platforms for maintaining cellular homeostasis and shaping neuroinflammatory signaling (18, 80, 103, 104). These non-fusogenic membrane contact sites form between the outer mitochondrial membrane and the lysosomal membrane; as with other contact sites, the intermembrane gap is typically ~10–30 nm, whereas MLCs average ~10 nm (103). The formation and dissolution of MLCs are governed by protein-based tethering/untethering machineries composed of proteins on both organelle membranes (105). **Table 1** summarizes the functional roles of MLCs-related proteins (**Table 1**). As a key environmental trigger for depression (106), chronic stress may influence the number and dynamics of MLCs primarily by perturbing mitochondrial and lysosomal function, rather than by directly damaging the contact structures (80, 104, 107, 108). Specifically, chronic stress can be accompanied by changes such as a decrease in mitochondrial membrane potential, restricted ATP production, increased ROS, and a decline in lysosomal acidification and degradation capacity (80, 108). At the molecular level, these stress-induced organelle disturbances can converge on key regulators of MLCs dynamics (103). These stress-associated alterations may shift Rab7 nucleotide cycling (18, 109, 110), reduce lysosomal Ca²^+^ release (e.g., TRPML1), and blunt TFEB-driven lysosome biogenesis (111–113). Collectively, these changes may reduce the number and stability of MLCs, weaken inter-organelle coordination, and contribute to early microglial phenotypes characterized by metabolic imbalance, impaired autophagic flux, and inflammatory priming (103, 104, 107). Beyond stress-related regulation, another unresolved issue concerns whether MLCs exhibit regional heterogeneity across different brain regions. Although direct evidence for brain region-specific differences in microglial MLCs is currently lacking, microglia are known to exhibit marked regional heterogeneity in their developmental trajectories and maturation, transcriptional identity, metabolic state, and inflammatory responsiveness (114–116). Brain regions such as the prefrontal cortex and hippocampus differ substantially in neuronal activity patterns, synaptic remodeling, and vulnerability to stress, all of which may influence microglial metabolic demands and organelle dynamics (117–120). Given that MLCs are highly sensitive to cellular metabolic and inflammatory states, it is plausible that microglial MLCs may exhibit brain region-dependent heterogeneity in composition or function (18, 81, 103). Elucidating such heterogeneity will require future brain region-specific analyses integrating microglia-specific purification techniques with ultrastructural analysis and proteomic profiling ultrastructural and proteomic approaches (18, 121).

**Table 1:** The functional roles of MLCs-related proteins.

Functional types	MLCs-related proteins	Abbreviation	Biological functions	Ref.
MLCs tethering & contact regulatory proteins	Ras-related protein 7	RAB7	Regulates MLCs contact site formation/dissociation via FIS1-TBC1D15-RAB7 axis	(18)
	TBC1 domain family member 15	TBC1D15	Mediates MLCs contact site assembly with FIS1	(197)
	Mitochondrial fission 1 protein	FIS1	Recruits TBC1D15 to modulate MLCs stability	(198)
	Vacuolar protein sorting 39	VPS39	Modulates autophagosome-lysosome formation at MLCs	(199, 200)
	Lysosome-associated membrane protein 1	LAMP1	Contributes to lysosomal membrane stability at MLCs	(201)
	Niemann-Pick type C1 protein	NPC1	Mediates cholesterol transport at MLCs	(202)
MLCs dynamic regulatory proteins	Optic atrophy 1	OPA1	Regulates mitochondrial fusion/cristae stability	(203)
	Dynamin-related protein 1	DRP1	Recruited to MLCs-associated mitochondrial fission sites and lysosomes-marked DRP1-dependent constriction sites	(204)
Calcium signaling & metabolic regulation proteins	Mucolipin 1	TRPML1	Mediates Ca²^+^ transfer to mitochondria	(81)
	Voltage-dependent anion channel 1	VDAC1	Influences mitochondrial Ca²^+^ handling, potentially interacting with lysosome-derived Ca²^+^ signals downstream of MLCs	(141)
	Mitochondrial calcium uniporter	MCU	Mediates Ca²^+^ uptake at MLCs	(205)
Autophagy-related proteins	PTEN-induced kinase 1	PINK1	Initiates MLCs-dependent mitophagy	(140)
Proteins related to inflammation and oxidative stress	Transcription factor EB	TFEB	Regulates lysosomal biogenesis through Ca²^+^ signaling	(206)
	Mechanistic target of rapamycin complex 1	mTORC1	Inhibits autophagy via MLCs-related energy signaling	(207)
	Microtubule-associated protein 1 light chain 3	LC3	Accumulates upon MLCs-impaired autophagosome-lysosome fusion	(208)
	Vacuolar ATPase subunit 1A	ATP6V1A	Regulates lysosomal acidification at MLCs and maintains autophagic flux	(80, 209)
	NLR family pyrin domain containing 3	NLRP3	Facilitate inflammasome activation	(210)
	Glucose-regulated protein 75	GRP75	Mediates mitochondria-lysosome contact and regulates MLCs-related oxidative stress	(211)
	NADPH oxidase 4	NOX4	Produces ROS via MLCs-related pathway as well as exacerbates MLCs dysfunction and oxidative stress	(212)
	Thioredoxin-interacting protein	TXNIP	Increases MLCs-related ROS and promotes NLRP3 activation	(191, 213)
	Sirtuin 3	SIRT3	Maintains the stability of MLCs and inhibits microglial inflammation	(214)
	Nuclear factor erythroid 2-related factor 2	Nrf2	Inhibits NLRP3 via HO-1 and regulates the MLCs-ROS anti-inflammatory pathway	(214, 215)

### 3.4 Microglial MLCs-mediated Ca²^+^ signal regulation

Microglial MLCs are crucial sites for Ca²^+^ inter-organelle transport and signal transduction, and their dysfunction can lead to Ca²^+^ signaling dysregulation, thereby exacerbating neuroinflammation and depressive pathology. Under physiological conditions, lysosomes release Ca²^+^ through the TRPML1 channels located on MLCs, and Ca²^+^ is quickly absorbed by mitochondria to promote ATP synthesis and autophagy activation. Furthermore, Ca²^+^ release mediated by TRPML1 promotes the nuclear translocation of TFEB, supporting lysosome formation and the expression of autophagy-related genes, thereby maintaining microglial homeostasis (122–125). However, under chronic stress, excessive ROS production damages the TRPML1 channels, reducing lysosomal Ca²^+^ release (123, 124, 126–128), which not only disrupts mitochondrial Ca²^+^ uptake, decreasing ATP production and impairing microglial metabolism but also inhibits TFEB nuclear translocation and lysosome regeneration (129). This dysregulation of Ca²^+^ signaling not only impairs the maintenance of microglial homeostasis but also disrupts neuronal synaptic plasticity, thereby further promoting neuroinflammation and ultimately exacerbating the development of depressive symptoms (130).

### 3.5 Microglial MLCs-mediated autophagic regulation

Microglial MLCs are critical regulatory sites for mitochondrial quality control and autophagic pathways. Their dysfunction can exacerbate neuroinflammation-induced depression by blocking autophagic flux (131). Under physiological conditions, stable MLCs regulate lysosomal acidification, enzymatic activity, and autophagosome maturation (132), ensuring effective recognition and clearance of damaged mitochondria to prevent inflammation caused by organelle accumulation. Meanwhile, MLCs maintain autophagic flux stability through the Ca²^+^–TFEB axis and AMPK/mTORC1 pathways (133–135). Chronic stress, which reduces MLCs number and stability, weakens the interaction between mitochondria and lysosomes (19, 124, 136). On the other hand, it blocks the fusion of autophagosomes and lysosomes, slowing autophagic flux and causing the accumulation of damaged organelles, protein aggregates, and lipids, which further exacerbates microglial stress (137–139). A decline in autophagic function further impairs mitochondrial quality control, exacerbates oxidative stress and neuroinflammation, and reduces synaptic plasticity (95), ultimately accelerating the progression of depression.

### 3.6 Microglial MLCs-mediated ROS feedback

Microglial MLCs serve as crucial hubs for ROS transmission, signaling amplification, and inflammatory activation. Dysfunction in these sites exacerbates neuroinflammation and depression through ROS-mediated signaling pathways. When MLCs become structurally unstable or reduced in number due to chronic stress, mitochondrial quality control is compromised, and the accumulation of defective mitochondria leads to excessive ROS (140, 141). As inter-organelle contact sites, MLCs amplify ROS signaling and act as crucial platforms for its transmission. On one hand, excessive ROS directly damages the TRPML1 channel (125) and the TFEB–mTORC1 pathway. On the other hand, mitochondrial ROS (mtROS) and mitochondrial DNA (mtDNA) act as danger signals, promoting NLRP3 inflammasome activation, which can accumulate at mitochondrial-associated membrane platforms (142). This cascade of reactions continuously impairs neuronal function and synaptic plasticity, ultimately driving the onset and progression of depression mediated by neuroinflammation.

In this context, a key unresolved issue is whether MLCs dysfunction in microglia is an initiating factor in the pathogenesis of depression or a secondary consequence of neuroinflammation. Current evidence supports a bidirectional and stage-dependent relationship rather than a strictly upstream or downstream causal model (143, 144). In the early stages of the disease or prior to symptom onset, mitochondrial stress, impaired lysosomal degradation capacity, and compromised organelle quality control may precede or occur concomitantly with neuroinflammatory escalation, thereby predisposing microglia toward a pro-inflammatory phenotype (145, 146). During this phase, destabilization of MLCs may promote mitochondrial ROS accumulation, impaired mitophagy, and calcium signaling imbalance, ultimately increasing the sensitivity of microglia to inflammatory stimuli and enhancing the pro-inflammatory signaling pathways (147, 148). Conversely, once neuroinflammation is initiated, persistent inflammatory cytokines, oxidative stress, and neuroendocrine stress responses can further disrupt mitochondrial dynamics and lysosomal function, leading to secondary damage to MLCs integrity and reinforcing the inflammatory cascades (149, 150). It is important to note that current temporally resolved studies in depression models are limited in definitively delineating the chronological sequence between MLCs disruption, microglial activation, and depressive-like symptoms (18, 81, 151). Synthesizing existing findings, it can be hypothesized that MLCs dysfunction in microglia contributes to a feed-forward pathogenic cycle, organelle stress and inflammatory responses mutually reinforce each other, sustaining neuroinflammation and increasing vulnerability to depression-related neuropathology (152, 153). Accordingly, MLCs impairment may exacerbate neuroinflammation through multiple mechanisms, and may promote the persistence and exacerbation of depression-related pathological processes.

## 4 The novel strategies of antidepressant therapy targeting MLCs

**Figure 1:**
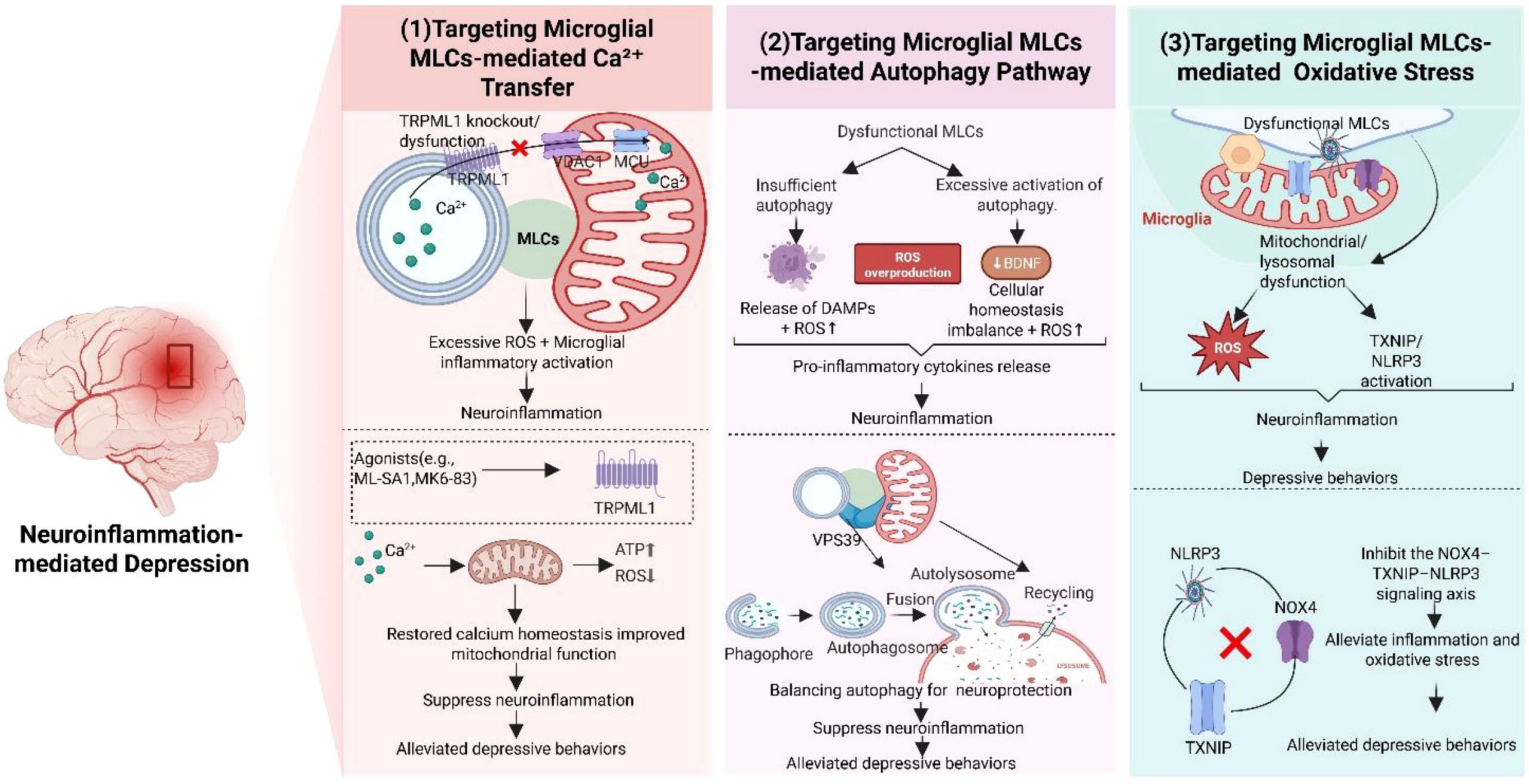
Targeting microglial MLCs – An innovative approach for treating neuroinflammation-associated depression. Multiple signaling pathways in microglial MLCs may represent new therapeutic targets for neuroinflammation-associated depression (1). During the development of neuroinflammation-associated depression, calcium signaling plays a key role in regulating mitochondrial function and neuronal activity. TRPML1, located on the lysosomal membrane, can modulate MLCs-mediated calcium transport; and its dysfunction leads to calcium homeostasis dysregulation. Using its agonists (ML-SA1, MK6-83) can restore calcium balance and mitochondrial function, thereby alleviating neuroinflammation and mitigating depressive symptoms (2). Autophagy exhibits dual functions in neuroinflammation. On one hand, it can maintain cellular homeostasis. On the other hand, insufficient autophagy activity causes the release of DAMPs and elevated ROS levels. Excessive autophagy activation results in reduced BDNF levels and cellular homeostasis imbalance, exacerbating neuroinflammation. At microglial MLCs, the core tethering protein VPS39 participates in regulating autophagic flux, and alleviating neuroinflammation through balancing autophagic activity, thereby mitigating depressive symptoms (3). In addition, oxidative stress pathways may also serve as potential therapeutic targets. The signaling axis formed by NOX4, TXNIP, and NLRP3 in the MLCs region is activated under stress, triggering ROS accumulation and TXNIP/NLRP3 activation, which induces neuroinflammation. Inhibiting this signaling axis can reduce neuroinflammation and alleviate depression. MLCs, Mitochondria-Lysosome Contact sites; TRPML1, Transient Receptor Potential Mucolipin 1; BDNF, Brain-Derived Neurotrophic Factor; VPS39, Vacuolar Protein Sorting-Associated Protein 39; NOX4, Nicotinamide Adenine Dinucleotide Phosphate Oxidase 4; TXNIP, Thioredoxin-Interacting Protein; NLRP3, NOD-like Receptor Pyrin Domain-Containing Protein 3; ROS, Reactive Oxygen Species.

Microglial MLCs act as sites for signal transfer and material exchange between mitochondria and lysosomes. Processes controlled by MLCs, such as Ca²^+^ signaling and autophagy control, provide potential novel molecular targets for neuroinflammation-associated depression (**Figure 1**).

### 4.1 Targeting microglial MLCs-mediated Ca²^+^ signal regulation

Microglial MLCs serve as critical hubs for interorganellar Ca²^+^ crosstalk. MLCs provide nanoscale proximity, spatially coupling lysosomal Ca²^+^ release (such as TRPML1) with mitochondrial uptake mechanisms, thereby promoting Ca²^+^ transfer and shaping metabolic and inflammatory phenotypes (81). Ca²^+^ disorder can be accompanied by a decrease in mitochondrial bioenergetics, and occurs concurrently with impaired lysosomal acidification and hydrolase activity (21, 154). These microglial dysfunctions further induce excessive ROS production and inflammatory activation, thereby exacerbating neuroinflammation and promoting depression-like phenotypes. TRPML1 is a key regulator of Ca²^+^ crosstalk at microglial MLCs. When TRPML1 is activated, it causes Ca²^+^ release from lysosomes and allows Ca²^+^ to enter mitochondria through MLCs. Jia-Wen Mo et al. reported that the lysosomal TFEB–TRPML1 axis in astrocytes modulates depressive-like behaviors, supporting a broader role of glial lysosomal Ca²^+^ signaling in affective regulation (111). In microglia, MCOLN1/TRPML1 deficiency is associated with a pro-inflammatory molecular signature and neuroinflammatory responses, indicating that impaired TRPML1-dependent lysosomal Ca²^+^ signaling can bias microglia toward inflammatory activation (155–157), which contributes to the progression of neuroinflammation-associated depression. Recent studies have shown that in depression models, certain drugs and molecules, such as ML-SA1 and MK6-83, have been tested in preclinical settings and shown to activate TRPML1, increasing Ca²^+^ transfer from lysosomes to mitochondria, potentially restoring the MLCs function and reducing inflammation (158–160). However, TRPML1 agonists such as ML-SA1 are not inherently microglia-specific and can also influence lysosomal Ca²^+^ signaling in neurons and other glial cell types (111, 161–163). In this context, current evidence suggests that microglia-specific modulation of MLCs is more likely to depend on targeted delivery strategies, rather than on the intrinsic pharmacological selectivity of available compounds. Possible approaches include exploiting microglia-enriched uptake pathways, e.g., through CSF1R-, TREM2-, CX3CR1-, or P2RY12-associated mechanisms (164–170), as well as cell- specific gene expression strategies employing microglia-specific promoters such as TMEM119 or P2RY12 for experimental validation and prospective gene therapy applications (171–180). Together, these targeted delivery and cell-specific gene expression strategies outline a conceptual framework for improving therapeutic precision while potentially limiting off-target effects. Nevertheless, rigorous safety assessment and *in vivo* validation remain essential, given the conserved and indispensable roles of lysosomal Ca²^+^ signaling and organelle contact sites across multiple CNS cell populations (162, 181, 182).

### 4.2 Targeting microglial MLCs-mediated autophagic regulation

Autophagy is a basic cellular process for maintaining cellular homeostasis and physiological balance (183). In microglia, efficient autophagy/lysosomal clearance supports mitochondrial quality control and limits the accumulation of mitochondrial-derived danger signals, thereby restraining ROS production and pro-inflammatory cytokine release and mitigating neuroinflammation-induced neuronal injury (184). Under chronic stress–related conditions, mitochondrial and lysosomal dysfunction may remodel MLCs dynamics and compromise lysosomal degradative capacity, contributing to impaired autophagic flux and the accumulation of damaged mitochondria and other toxic substrates, which in turn biases microglia toward inflammatory activation and neuroinflammation amplification (104, 185). However, translating these MLCs-related therapies into clinical practice faces several challenges. Li et al. summarized that VPS39, as a HOPS/tethering-related factor, regulates autophagosome–lysosome fusion and may influence mitochondria–lysosome functional coupling, providing a plausible molecular link between MLCs-associated organelle coordination and autophagic flux control (186). Persistent microglial autophagy dysregulation (including flux impairment and/or maladaptive activation depending on context) can further reinforce oxidative stress and pro-inflammatory signaling cascades such as NLRP3 inflammasome pathways, thereby supporting neuroinflammation-driven depressive pathophysiology (187).

### 4.3 Targeting microglial MLCs-mediated redox homeostasis

Besides regulating Ca²^+^ signaling and autophagy, emerging evidence shows that microglial MLCs dysfunction may influence redox homeostasis and neuroinflammation. Under stress-related conditions, mitochondrial dysfunction and lysosomal impairment in microglia are frequently accompanied by increased oxidative stress and inflammatory signaling, including activation of the TXNIP–NLRP3 inflammasome axis. TXNIP can translocate to mitochondria under cellular stress and has been implicated in promoting NLRP3 inflammasome activation (188), while NOX4-derived ROS may further enhance TXNIP induction, thereby amplifying inflammatory signaling (189–191). Importantly, TSPO is closely associated with microglial inflammatory states, and TSPO ligands have been reported to suppress NLRP3-related inflammation in microglial models, potentially through improving mitochondrial quality control (192, 193). Although direct nanoscale localization of NOX4/TXNIP/NLRP3 specifically at MLCs microdomains remains limited in the depression context, these pathways converge on mitochondria- and lysosome-dependent stress responses—processes that are functionally coupled to MLCs dynamics. Therefore, targeting MLCs-regulated organelle coordination may indirectly attenuate oxidative stress–inflammasome amplification and alleviate neuroinflammation-driven depressive phenotypes (194–196).

## 5 Summary and outlook

In recent years, there has been growing interest in intracellular signaling, particularly the crosstalk between different organelles. MLCs orchestrate the maintenance of cellular physiological homeostasis in microglial cells and engage in crosstalk with multiple signaling cascades related to neuroinflammation. However, the role of MLCs in microglia in depression remains underexplored. More studies are now focusing on organelle movement, autophagy, and metabolic signaling related to MLCs in microglial cells, showing their potential value in identifying new treatment targets for depression. To fill the gap in our understanding of MLCs’ role in microglia and depression, future research should focus on elucidating how MLCs dysfunction in microglia specifically contributes to neuroinflammation and depressive pathology. While therapeutic strategies for depression remain limited, targeting MLCs-related signaling pathways in microglial cells may offer novel therapeutic approaches for this complex disorder.
